# AceTree: a major update and case study in the long term maintenance of open-source scientific software

**DOI:** 10.1186/s12859-018-2127-0

**Published:** 2018-04-04

**Authors:** Braden Katzman, Doris Tang, Anthony Santella, Zhirong Bao

**Affiliations:** Developmental Biology Program, Sloan-Kettering Institute, New York, NY USA

**Keywords:** *C. elegans*, 4D, 3D, Fluorescence microscopy, Automated lineaging, Embryogenesis, Affine transformation, Interface

## Abstract

**Background:**

AceTree, a software application first released in 2006, facilitates exploration, curation and editing of tracked *C. elegans* nuclei in 4-dimensional (4D) fluorescence microscopy datasets. Since its initial release, AceTree has been continuously used to interact with, edit and interpret *C. elegans* lineage data. In its 11 year lifetime, AceTree has been periodically updated to meet the technical and research demands of its community of users. This paper presents the newest iteration of AceTree which contains extensive updates, demonstrates the new applicability of AceTree in other developmental contexts, and presents its evolutionary software development paradigm as a viable model for maintaining scientific software.

**Results:**

Large scale updates have been made to the user interface for an improved user experience. Tools have been grouped according to functionality and obsolete methods have been removed. Internal requirements have been changed that enable greater flexibility of use both in *C. elegans* contexts and in other model organisms. Additionally, the original 3-dimensional (3D) viewing window has been completely reimplemented. The new window provides a new suite of tools for data exploration.

**Conclusion:**

By responding to technical advancements and research demands, AceTree has remained a useful tool for scientific research for over a decade. The updates made to the codebase have extended AceTree’s applicability beyond its initial use in *C. elegans* and enabled its usage with other model organisms. The evolution of AceTree demonstrates a viable model for maintaining scientific software over long periods of time.

## Background

The invariant lineage of the nematode *C. elegans* [1] makes the organism a powerful model for studying developmental processes. StarryNite, a software package released in 2006, performs automated lineage extraction by segmenting and tracking fluorescently labeled nuclei in 4D microscopy datasets [2]. AceTree, a companion program built to view and edit the nuclear tracking data generated by StarryNite, facilitates interpretation validation and quality control of StarryNite results [3].

AceTree, developed beginning in 2005, has since its initial release provided a comprehensive set of tools for interacting with lineage data, both in a 2-dimensional (2D) viewing window where tracks are superimposed on nuclear images and as an abstracted lineage tree [4]. Users can explore their data both in time and space, by moving up and down within and between annotated image stacks. Additionally, a 3-dimensional viewing window provides an abstract view of nuclear positions as a cloud of 3D spheres. This representation of the data provides a more intuitive sense of the positions of cell bodies in space than can easily be achieved by moving between 2-dimensional image slices.

Continuously in use for the 11 years since its initial release, AceTree has been periodically updated to meet the technical and research demands of its community of users. The software has proved to be a useful tool in research, necessitating evolutionary changes as software libraries and microscopy technology have evolved.

AceTree’s latest release provides a multitude of changes aimed at meeting the demands of its community and incorporates new features for visualization and analysis. A large-scale user-interface update adds new tools, removes obsolete ones and facilitates improved accessibility of key functionality. A revised image loading pipeline supports greater flexibility in input images. Revisions to canonical name assignment allow for the free orientation of embryos in 3-dimensional space and an entirely new 3-dimensional viewing window provides a new suite of methods for exploring cell positions.

### Related software

When AceTree was first released, its primary competitors were Simi BioCell and Angler. Simi BioCell, a commercial product that enables tracking and documenting cellular divisions, is still aimed at manual lineaging [5], a significant disadvantage to the automated lineaging pipeline in AceTree. Angler, a companion program to the AceDB database that facilitates visualization of DIC (differential interference contrast) microscopy images coupled with lineage data and 3D cell positions [6], lacks the ability to edit annotation data as is possible in AceTree.

A number of other related software packages and tools have been released since the initial AceTree release that contain similar image analysis, cell lineaging and editing tools. These tools are, for the most part, optimized for managing large datasets and emphasize visualization. The Imaris for Cell Biologists software package contains organism agnostic modules for tracking cell divisions and recording lineages, distributed as a commercial product [7]. In the open-source scientific software community, LEVER and CloneView, VAA3D (3D Visualization-Assisted Analysis), Endrov, and the visualization and lineage curation tool developed by the Keller Lab are worthy of discussion based on their shared functionality with AceTree [8–11].

LEVER (Lineage Editing and Validation), an image analysis, curation and visualization suite that tracks and analyzes dividing stem cells in large microscopy datasets, automatically generates a lineage tree of clones during cell proliferation. It contains similar editing tools to AceTree and is paired with a powerful web visualization tool called CloneView, but it is limited to 2D image series [8]. VAA3D is a visualization focused software suite that contains analysis modules for neuron tracing which resemble AceTree’s manual curation functionality in 4D image series [9]. Endrov, an image-analysis program last updated in 2013, contains much of the same tracing and lineaging functionality as AceTree, enabling annotation in two and three dimensions [10]. The Keller Lab’s 2014 publication on lineage reconstruction describes a software suite similar to the StarryNite and AceTree suite that they developed to reconstruct cell lineages in large fluorescence microscopy data [11]. The relevant lineage curation and editing tools of their pipeline share the same functionality as AceTree while being optimized for large data sets, though they lack the worm specific features.

While there have been major strides in visualization and lineaging software over the last 10 years, we believe AceTree remains a reliable option for use in embryonic contexts when cell lineaging and manual curation is necessary. AceTree has a history of being used for fully editing large numbers of embryonic lineages, and it is not clear how many of the programs discussed above would scale to complete curation in the *C. elegans* lineage. Because of its ongoing usage in these contexts for a decade and its special focus on carrying out lineaging and editing tasks, AceTree is the most robustly tested and reliable solution for the embryonic worm.

## Implementation

AceTree is written in Java, and has been updated to Java 1.8 to allow the use of new language and library features and remove dependencies on deprecated libraries. AceTree’s new 3-dimensional visualization window, derived from the WormGUIDES atlas [12], is written in Java using the JavaFX 8 platform. Development of the software is carried out in the open-source IntelliJ integrated development environment (IDE). The program is packaged as a cross platform JAR (Java Archive) file and has been tested on Linux (Ubuntu 14.04, 16.04), Windows (7 Professional, 10) and macOS (10.13 High Sierra).

Github provides source code and instructions for development setup: https://github.com/zhirongbaolab/AceTree.

## Results

### Interface

**Fig. 1:**
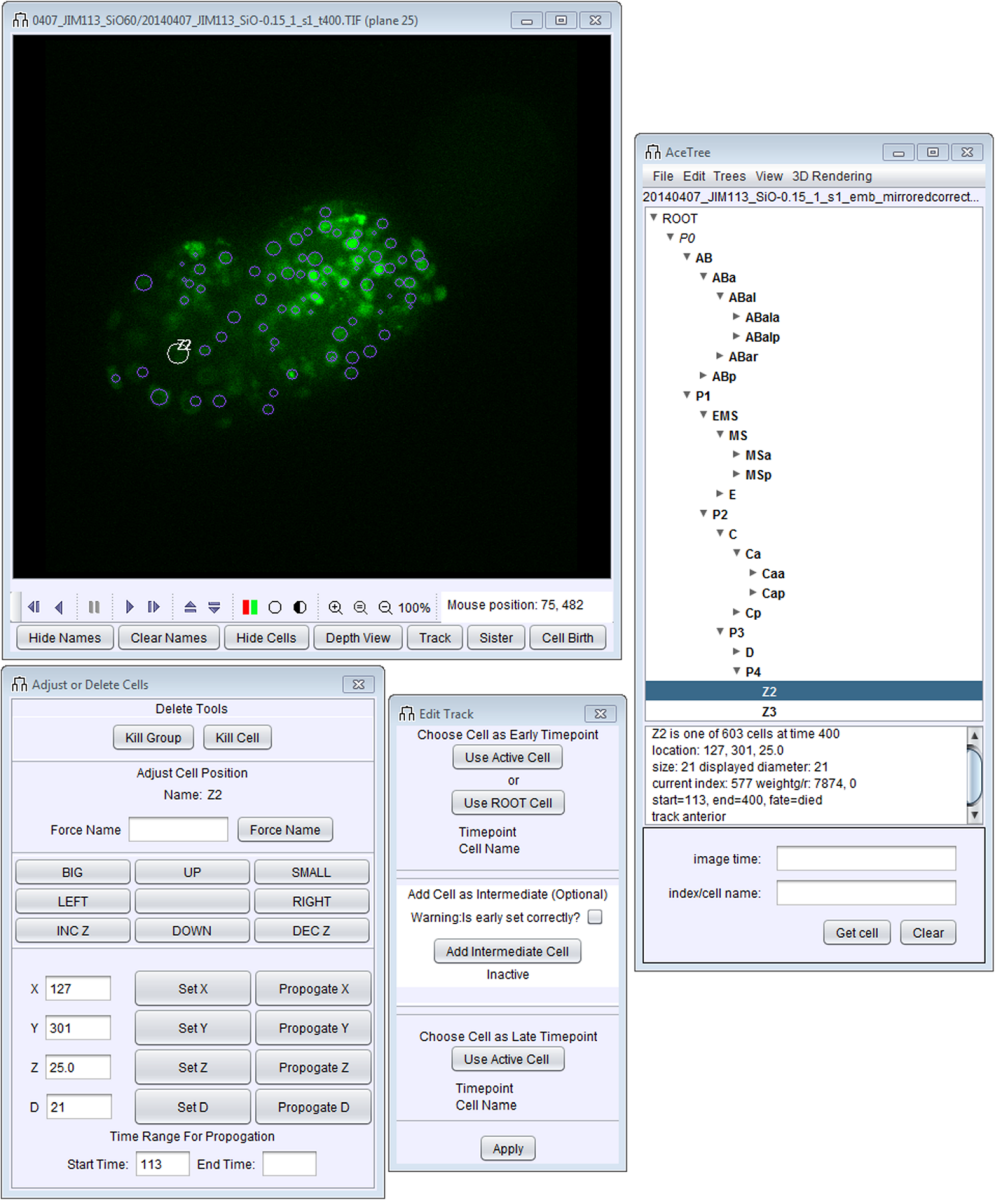
An overview of the revised AceTree user interface. All display related functionality and commonly used toggles that control the appearance of the image window (time, plane, color channels, zoom, labels, tracking) are now in the image window (top left). Cell editing tools (bottom left) and track editing tools (bottom right) have been grouped in separate windows to better organize tools while enabling individual users to create their own preferred layout

The user-interface has been rearranged to better organize tools, grouping features with shared purposes together when possible, see Fig. 1. Viewing controls such as time and plane, color channel selection and controls for cell selection and labeling have been moved to the image window in order to concentrate display controls in a toolbar within the main 2D image window. Editing tools have been reorganized, placing manual tracking and track editing tools together. The file menu has been updated by grouping functionality more systematically and removing obsolete tools.

The rearranged user-interface also integrates new image controls. The image window now includes zoom and brightness levels controls.

### Flexibility

A collection of changes have been made to increase the flexibility and usefulness of AceTree in a variety of developmental contexts. Later stages of *C. elegans* embryonic development are increasingly accessible due to advances in imaging and techniques for computationally untwisting embryos after muscular twitching begins [13]. In toto imaging of other organisms is also increasingly possible [14, 15] while navigating and interpreting large datasets remains challenging. New AceTree features address previous limitations and benefit the *C. elegans* research community while in many cases also increasing AceTree’s usability with other model organisms.

Functional name data from the *C. elegans* Parts List [1] has been fully integrated into AceTree. Search functionality throughout uses functional and systematic names interchangeably. This extension is useful later in embryonic development as terminal cells can be more easily recognized by their functional names.

Systematic name assignment code has always been built into AceTree. Originally, name assignment was manually rerun when users needed to update naming during tree edits. Now, name assignment is automatically updated with every user edit to the lineage.

AceTree first supported naming only on canonically oriented embryos. Later functionality was added to allow the naming of randomly positioned embryos, removing the need to orient embryos canonically on the slide or in post-processing. However, the assumption remained that embryos were mounted compressed [3]. With this mounting method the Left-Right (LR) axis of the 4-cell stage embryo aligns with the axial direction. Though this mounting is convenient in many circumstances, it is often desirable to image the embryo from different orientations in order to better observe specific structures. Additionally, new imaging approaches, such as the dual-view inverted selective plane illumination microscope (diSPIM) [16], require an uncompressed mount, meaning embryos can be rotated randomly around their Anterior-Posterior (AP) axis. To support naming in these contexts, a new, optional naming mode has been introduced in which the AP and LR vectors of the 4-cell embryo are directly specified. These values are used to translate between image and canonical embryo space, allowing embryos to be named even when arbitrarily oriented in 3D. Two caveats remain, expected division orientation vectors are still based on data from compressed embryos, and in some cases division axes can be significantly different relative to the body axes under the two mounting conditions, resulting in an increased rate of naming errors. In addition, expected division axes are missing for many tenth round divisions. Naming in these cases continues to revert to default body axis based naming. Collecting empirical division axis expectations for the tenth round and in uncompressed embryos remains future work.

**Fig. 2:**
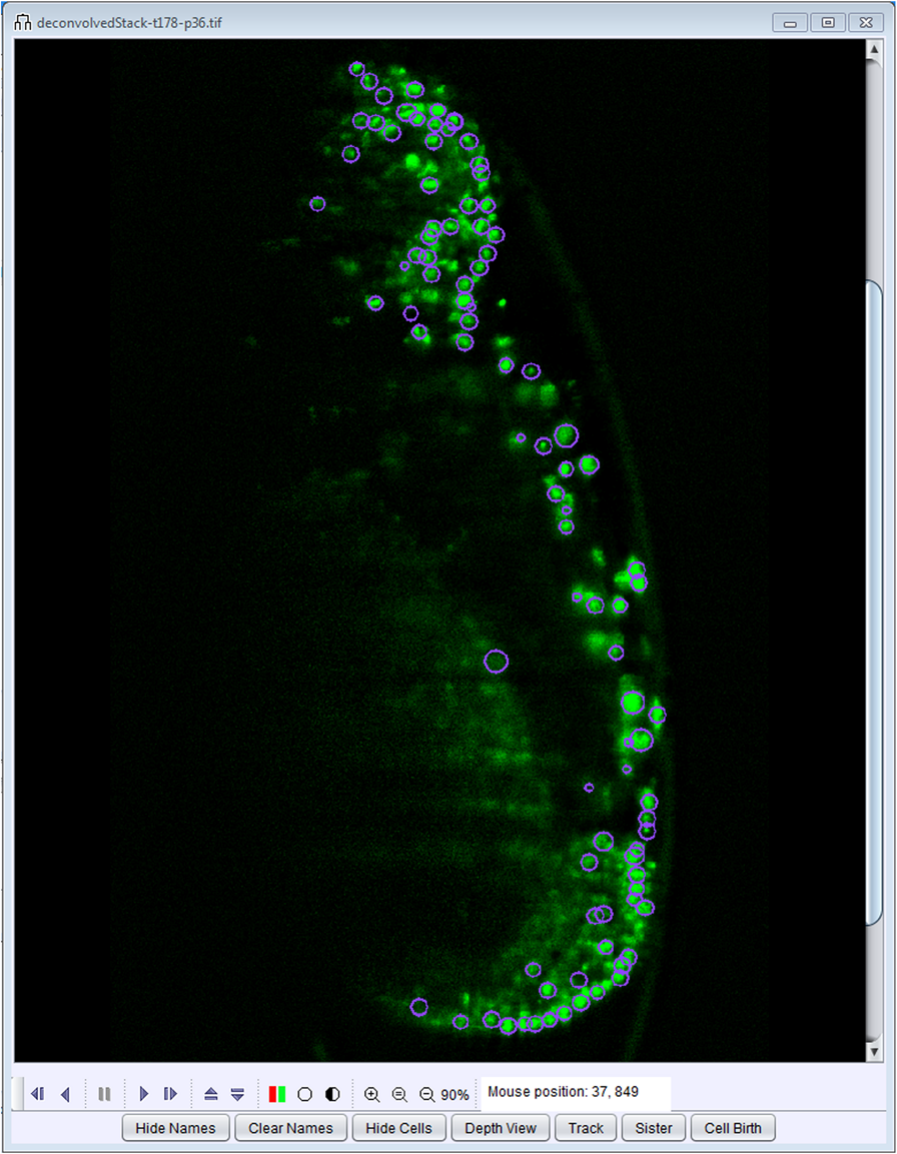
A drosophila embryo in AceTree [14]. AceTree can support interpreting and lineaging for large datasets using optimized loading and editing methods and a generalization of the force naming tool

Fluorescence microscopy has evolved enormously in the past decade. New techniques have enabled complete imaging in larger organisms like drosophila and zebrafish [14, 15] with larger image volumes, longer developmental times and tens of thousands, instead of hundreds, of cells. In light of these advancements, AceTree has been extended to support longer movies and higher cell counts. Restrictions on maximum slices and frames have been removed and loading and updating internal data structures has been optimized to allow much larger files to be effectively loaded and edited. Names can now be manually assigned to any cell, even when no *C. elegans* embryo is detected, allowing completely manual naming to be used when desired. This collection of functionality simplifies the use of AceTree for other model organisms, see Fig. 2. For example, Keller et al. used AceTree on partially tracked, completely unedited *Drosophila* embryos as a quality control tool in their creation of a fly digital embryo [17]. To run quality control on *Drosophila* segmentation data, the study relied on AceTree as an interactive tool for parameter tuning. A second illuminating example of AceTree’s use in other organisms is the Takashi Hiirage Group’s research into epithelial polarity in the early mouse embryo where powerful lineaging and editing tools were sought. To examine the dynamics of Cdx2 protein expression in a Cdx2-EGFP x H2B-mCherry mouse embryo, nuclei were tracked and lineaged using the StarryNite and AceTree suite [18]. AceTree was used in this study to trace and examine lineage segregation in the early mouse embryo.

Lastly, AceTree was originally developed to work with 8bit images, but greater bit depth is currently available from most sensors. AceTree has been extended to read 16bit images and dynamically map them to display depth using interactive black and white point controls for each channel.

### 3D window

Many users find it challenging to build up a mental image of the 3D relative positions of objects by moving through an image stack. Often, it is easier to understand the relative position of nuclei in an abstract 3D model. This has made the 3D window an important AceTree feature from its first release. Initially, this window was implemented in Java3D, a high-level scene graph API (Application Programming Interface) for JAVA. Since then, Java3D has become a community source project, no longer directly supported by Oracle [19]. JavaFX is now the regularly maintained, integrated, high level 3D graphics library of the Java Runtime Environment and Java Development Kit (JRE, JDK) [20].

**Fig. 3:**
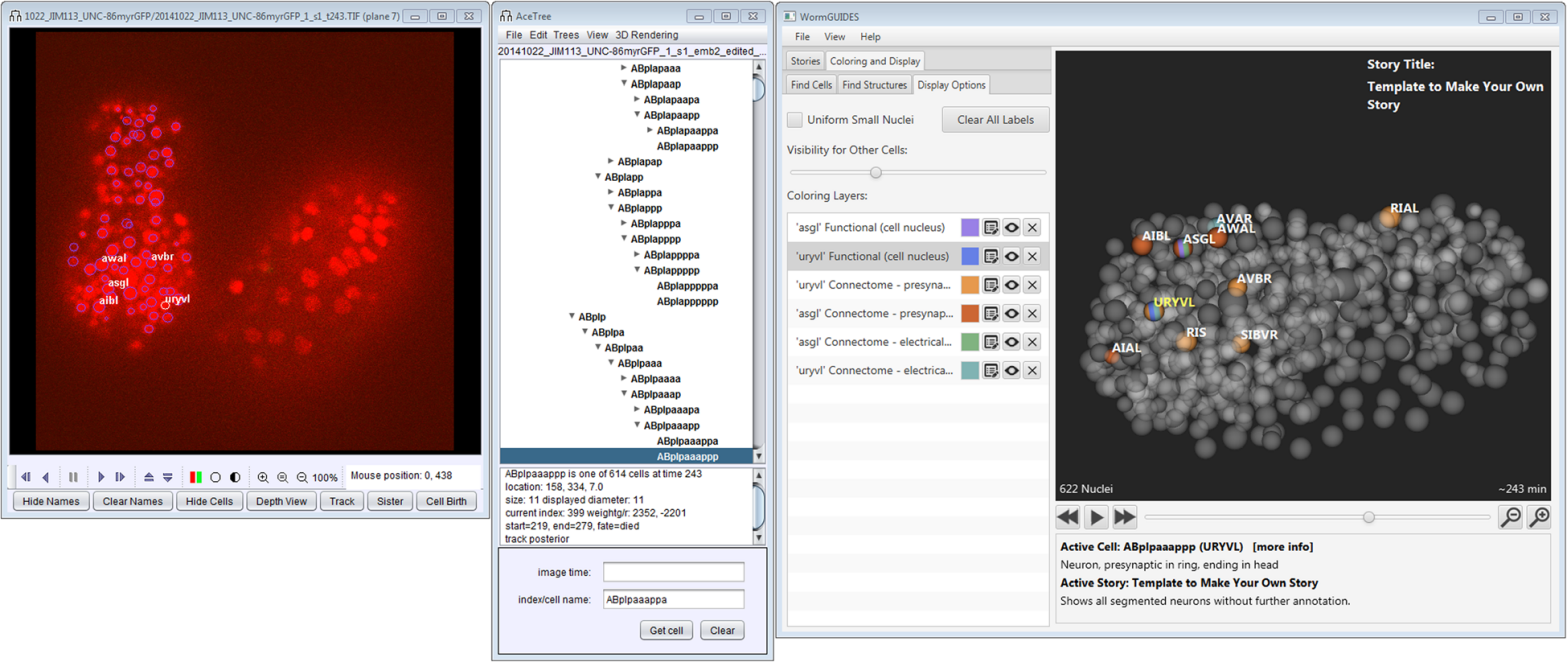
An overview of the new 3-dimensional viewing window. Rules can color cells based on a broad array of search criteria including adult neuronal connectivity. The ‘Coloring Layers’ show the presynaptic and electrical connections of the amphid neuron ASGL and the head neuron URYVL. Color striping indicates that multiple rules apply to the striped entity. Here, the stripes on ASGL and URYVL indicate the wiring relationships between them in the adult. The ‘Display Options’ tab provides a key for the model annotations (right). Other searched criteria that can be used include lineage name, functional name, ancestry, and gene expression

Lack of support means that Java3D is difficult to install and has not functioned on macOS platforms for some time. To address these deprecations, an entirely new 3D window for browsing the embryo was built in the context of the WormGUIDES neurodevelopmental atlas [12]. Built in JavaFX, this 3D window has been integrated into AceTree to serve as a replacement for the original 3D window, see Fig. 3.

In addition to a 3D display with controls, this viewer provides a new search interface for data exploration. Users can search for cells and color the nuclear position model by lineage name, functional name, Parts List [1] description, connectome, gene expression and ancestry.

## Discussion/conclusion

AceTree has undergone serious revisions in its 11 year lifetime. Its main windows have been largely reorganized and its internal representations extended and generalized. At this point, much of its core functionality has been either greatly extended or entirely rewritten from its initial state.

The continuous evolution of the AceTree software package is an intriguing case study in maintaining actively used scientific software. For over a decade, AceTree has been an important tool for scientific research in developmental biology labs, and has continually evolved to meet technology and research demands.

Typically, software is maintained in two ways, either by a team of dedicated developers in a commercial or infrastructure grant context, or by large scale open-source community efforts. Given its relatively modest but dedicated user base, AceTree has been maintained differently, with a small group of primary developers intermittently working on AceTree at different times during its lifetime. The changes that AceTree has undergone are a product of feedback from its community of users and changes in the software packages that it utilizes.

AceTree is not a heavily funded effort with full time staffers. Rather, AceTree has been maintained over a long period of time by a small circle of core labs that it serves. Maintenance is fueled by researchers who use it, incentivizing its continued availability and application in the community. Often, scientific software is released with the intention of ongoing use and adaptation by the open-source community. In reality, many of these projects are released and never used. AceTree’s continued usage and its responsiveness to the community demonstrate a model for how scientific software can work in the ever changing dynamics of the open-source user community.

AceTree’s development model works by periodically setting long term development goals that require significant developer time. By identifying predictable changes in software APIs, microscopy hardware and research contexts likely to arise in 1 year to 2 year timeframes, we set large development goals to be carried out as changes took place. The redesign of the user-interface to better organize tools and streamline the interface and the creation of a completely new 3D window, as described above, were the most significant of the long term goals. Proactively identifying these goals allowed planning for the developer time needed to ensure that AceTree would continue to be a useful tool.

Given this long term model of development, it was possible to plan when it would be necessary to maintain a dedicated part time developer for AceTree to complete these larger tasks and when maintenance could be performed by a postdoc in the interim periods. Always having someone familiar with the code base, even if they did not devote significant hours to it for long periods of time, ensured that unpredictable changes did not make AceTree unusable or obsolete. The most prominent examples of these unplanned, incremental changes are the iterative updates made to the image loading pipeline discussed above. These changes resulted from new collaborations and contexts that exposed unpredicted usage cases. As a result of maintaining a lab member who was always in a position to modify the codebase, supplemented by a developer when needed, AceTree evolved and remains a useful tool. AceTree’s development model demonstrates that a niche tool can driven by low level, ongoing, and intermittent focused development over a relatively long time frame.

We believe that the success and continued utility of AceTree establishes its evolutionary software development paradigm as a viable path for niche open-source scientific software. By proactively identifying development updates to be completed over longer periods and maintaining at least minimal development ability in house at all times, open-source scientific software can evolve with the predictable changes in research contexts, and be well positioned to respond to unforeseen changes. We felt it compelling to present this release of AceTree and its development model both because the updates significantly widen the possible community of users, and as an example of the practical concerns encountered when maintaining a fairly complicated code base over a decade timescale with limited developer resources.

## Methods

Some of the new features available in the software required building interfaces between old and new code. Two main interfaces are worthy of detailed description. First, in order to maintain the original lineage naming paradigm yet allow users to lineage uncompressed embryos, we created a new method for transforming an uncompressed embryo’s orientation to the expected canonical orientation. Second, to utilize AceTree’s internal data representation in the context of the 3D window built for WormGUIDES, we created an abstract interface for representing the underlying lineage data that adheres to the StarryNite model specification.

To support uncompressed reorientation, we created the CanonicalTransform class to transform any orientation supplied by the user to the canonical orientation of *C. elegans* (anterior to the left and dorsal up) [1], an internal requirement of AceTree for lineage naming as division expectations are stored in a canonical coordinate system. The user defines the 3-dimensional orientation of the embryo by supplying two vectors, AP and LR, in the metadata AuxInfo_v2.xml file. The CanonicalTransform class finds the transform from these vectors to their canonical orientations by computing the axis-angle representation of the transform [21]. The transform calculation includes the special degenerate cases of the axis-angle representation when the supplied axis is already canonical or flipped-canonical i.e. collinear. The two resulting transformation matrices, AP and LR, are then concatenated to create a single, affine transformation. This transform is then applied to all division axes before they are propagated to existing naming code which assigns lineage names based on the direction of these divisions in a canonical orientation.

To interact with the AceTree data representation in a WormGUIDES context, we created the NucleiMgrAdapter class to package AceTree’s data orderly and efficiently. The NucleiMgrAdapter class in AceTree’s source code implements the LineageData interface defined in the WormGUIDES package. This adapter bundles AceTree’s internal representation of the nuclei files, defined in the NucleiMgr class, into a form interpretable by WormGUIDES via the LineageData interface. This adapter is used to instantiate a WormGUIDES application instance in the WormGUIDESWindow class on a dedicated thread.

## Availability and requirements

Project Name: AceTree.

Project Home Page: https://github.com/zhirongbaolab/AceTree.

Operating Systems: Linux, Windows, macOS.

Programming Language: Java.

Other requirements: JRE 1.8 or higher.

License: GNU GPL.

Any restriction to use by non-academics: None.

## Abbreviations

4D: 4-dimensional
3D: 3-dimensional
DIC: ᅟ
2D: 2-dimensional
LR: ᅟ
AP: ᅟ
IDE: ᅟ
API: ᅟ
JAR: ᅟ
JRE: ᅟ
JDK: ᅟ
